# Supplementary material for: Charge-driven condensation of RNA and proteins suggests broad role of phase separation in cytoplasmic environments
Source: eLife. 2021 Jan 26;10:e64004. doi: 10.7554/eLife.64004 (PMC7877912; doi:10.7554/eLife.64004)
Supplement: Transparent reporting form [file elife-64004-transrepform.pdf]

eLIFE
1st Floor
24 Hills Road
Cambridge CB2 1JP, UK
P 01223 855340
W elifesciences.org
T @elife

eLife's transparent reporting form

We encourage authors to provide detailed information *within their submission* to facilitate the interpretation and replication of experiments. Authors can upload supporting documentation to indicate the use of appropriate reporting guidelines for health-related research (see EQUATOR Network), life science research (see the BioSharing Information Resource), or the ARRIVE guidelines for reporting work involving animal research. Where applicable, authors should refer to any relevant reporting standards documents in this form.

If you have any questions, please consult our Journal Policies and/or contact us: editorial@elifesciences.org.

Sample-size estimation

- You should state whether an appropriate sample size was computed when the study was being designed
- You should state the statistical method of sample size computation and any required assumptions
- If no explicit power analysis was used, you should describe how you decided what sample (replicate) size (number) to use

Please outline where this information can be found within the submission (e.g., sections or figure legends), or explain why this information doesn't apply to your submission:

The *in vitro* data observes condensation on the molecular level, which by its nature has very large number of samples. There are no animal or clinical data in this paper.

Replicates

- You should report how often each experiment was performed
- You should include a definition of biological versus technical replication
- The data obtained should be provided and sufficient information should be provided to indicate the number of independent biological and/or technical replicates
- If you encountered any outliers, you should describe how these were handled
- Criteria for exclusion/inclusion of data should be clearly stated
- High-throughput sequence data should be uploaded before submission, with a private link for reviewers provided (these are available from both GEO and ArrayExpress)

Please outline where this information can be found within the submission (e.g., sections or figure legends), or explain why this information doesn't apply to your submission:

Most data in Fig. 4 were measured once, although each measurement observed dozens or hundreds of condensates. Measurements in Fig. 5 were repeated 3 times, as shown in Supplementary Fisg. 26 and 27. Most data in Fig. 6 were measured once but one concentration was measured twice, once exhibiting phase separation and once not exhibiting phase separation. This indicates the reproducibility of creating the concentrations at the boundary between the phases. Data in Fig. 7 were measured once, except for one set of concentrations, indicated by two points, which shows the typical reproducibility.

Individual simulations were not repeated but a wide range of concentrations and conditions were considered, and simulations were extended well beyond typical times to achieve convergence. Moreover, highly consistent results were obtained for similar conditions indicating reproducibility of the results reported here (see for example Figure 2).

Simulation results reported based on particle averages (such as diffusion estimates) benefit from ensemble averages over tens to hundreds of equivalent particles.

Statistical reporting

- Statistical analysis methods should be described and justified
- Raw data should be presented in figures whenever informative to do so (typically when N per group is less than 10)
- For each experiment, you should identify the statistical tests used, exact values of N, definitions of center, methods of multiple test correction, and dispersion and precision measures (e.g., mean, median, SD, SEM, confidence intervals; and, for the major substantive results, a measure of effect size (e.g., Pearson's r, Cohen's d)
- Report exact p-values wherever possible alongside the summary statistics and 95% confidence intervals. These should be reported for all key questions and not only when the p-value is less than 0.05.

Please outline where this information can be found within the submission (e.g., sections or figure legends), or explain why this information doesn't apply to your submission:

The statistical analyses described above do not apply to most of the data in this submission for the reasons described above.

For reported results where statistical analyses are appropriate (such as particle-averaged diffusion estimates from simulation) standard errors of the mean are reported. P-values were not calculated because reported values inside and outside the condensates were grossly different compared to the estimated uncertainties.

(For large datasets, or papers with a very large number of statistical tests, you may upload a single table file with tests, Ns, etc., with reference to sections in the manuscript.)

Group allocation

eLife Sciences Publications, Ltd is a limited liability non-profit non-stock corporation incorporated in the State of Delaware, USA, with company number 5030732, and is registered in the UK with company number FC030576 and branch number BR015634 at the address 1st Floor, 24 Hills Road, Cambridge CB2 1JP | August 2014

- Indicate how samples were allocated into experimental groups (in the case of clinical studies, please specify allocation to treatment method); if randomization was used, please also state if restricted randomization was applied
- Indicate if masking was used during group allocation, data collection and/or data analysis

Please outline where this information can be found within the submission (e.g., sections or figure legends), or explain why this information doesn't apply to your submission:

This information does not apply to our submission due to the reasons indicated above.

Additional data files ("source data")

- We encourage you to upload relevant additional data files, such as numerical data that are represented as a graph in a figure, or as a summary table
- Where provided, these should be in the most useful format, and they can be uploaded as "Source data" files linked to a main figure or table
- Include model definition files including the full list of parameters used
- Include code used for data analysis (e.g., R, MatLab)
- Avoid stating that data files are "available upon request"

Please indicate the figures or tables for which source data files have been provided:

Quantitatively accurate results are reported in the text. Figures show qualitatively meaningful results and high-resolution image data is provided as supplementary data.
